# Book Reviews

**Published:** 1810-10

**Authors:** 


### CRITICAL ANALYSIS

OF

### RECENT PUBLICATIONS

IN THE

DIFFERENT BRANCHES OF PHYSIC, SURGERY, AMD MEDICAL PHILOSOPHY,

---

*Analysis of a Treatise on the Plica Polonica, to which are subjoined numerous Cases of that Disease.* By F. DE LA FONTAINE, first Surgeon to the late King of Poland. Paris, 1809. Drawn up by M. ROUSILLE CHAMSERU.

THE body of M. de la Fontaine's work consists of about 120 pages, one-third of which is devoted to a description of the endemic affection; a fourth part is filled with details as to the causes, and the rest consists of cases. The author promises in his preamble to conform to the motto, which he has borrowed from Selle: "*Theories were long in vogue; this is now the reign of observation.*" This oracle of a judicious physician recals the precept of the first master of the art, who requires that before attempting to reason, we should have acquired an intimate knowledge of the events, phænomena, and circumstances, which form a disease. It is the part of nature, when well examined, to enlighten the mind: f, instead of setting out from palpable facts, reason takes hold of probable suppositions, the medical consequences may be dangerous. Such is the precept of Hippocrates as to the true mode of observation to be practised, and which no modern, in my humble opinion, has followed with more precision than Morton.

M. de la Fontaine affirms what is at best doubtful, when he says, that the plica is occasioned by a subtle matter, which is thrown in a critical manner on the hair: In a first definition we only require the sensible aspect of the thing, and a faithful image of the injured organs, without mixing the smallest probable supposition of a reason too apt to mislead. "The plica spares neither age, sex, nor rank; and strangers, when newly arrived in Poland, are liable to it as well as the natives." This assertion is exaggerated in all its bearings: so far as strangers are concerned, the long stay of the grand army, in the plains of the Vistula, give the most formal contradiction to the assertion on this head.

Far less do I understand his doctrine of contagion—"The first species of contagion, the most common, and that which produces the greatest number of cases of plica, is that which has been brought into the world with the child." I ask if this ought to be called contagion? Is it not rather a disposition, a hereditary transmission, which also requires its proof? The second kind of contagion takes place, because the disease has been "communicated by the nurse, or by some person who was attacked by it and with whom we may have slept." It is admitted, however, that the plica is rarely contracted in this manner. He also conjectures, that the virus may be received from a woman with whom a person has intercourse, whether she has the matter of the plica still lurking in the mass of humours, or even has the disease in the "genital parts;" but to these vain suppositions I have to oppose innumerable examples of married couples, children, and animals, crowded together under one roof, and yet the disease has been fortunately confined to one individual.

A third kind of contagion proceeds from "the clothes, such as hats, caps, &c. which have been worn by several persons." This, the author informs us, is the least violent of all; it, however, brings some tales to his recollection, which I cannot conscientiously regard as matters of fact; in this manner setting out from a primary species of contagion, which really does not exist, the two succeeding kinds go on diminishing, till the object is reduced to nothing, and the phantom vanishes.

M. de la Fontaine describes at great length, the symptoms which he ascribes to plica; this part which has been treated by so many authors, ought to have been limited to a support of the diagnostic of the precise assemblage of symptoms peculiar to a lesion *sui generis*, instead of encumbering with the special symptoms of an endemic every imaginable disease. No one can now be ignorant that we ought to be on our guard against these prolix and unconnected descriptions, surcharged with superannuated theories, humours, morbid matters, lymph, &c. and in which more than doubtful observations are drowned in a deluge of words. Persons have not scrupled to assimilate the plica to the plague, and to syphilis, in order to give countenance to the hypothesis of one and the same degree of virulence and contagion, to the small pox, so deduce a similar affection as inherent in Polish blood—to leprous diseases, with which, what I may call the leprosy of Poland, has some analogy in its origin, principle, and phenomena. This last comparison is the only plausible one, in my opinion, the cessation of the lepra in Europe, having been marked by circumstances capable of enlightening enlightening our medical police, as to the true methods of extirpating the *trichoma*.

The mania for scholastic distinctions, even in the present stage of our analytical knowledge, is a reproach to us; pathology has been already purged from it in a few good modern works; and when I reflect on the nosographical surveys of Pinel and Richerend, I see in them a reciprocal emulation of a good analytical method, in the two first branches of medical science. The plica is unique in its seat, in its principle; and every thing connected with the wretched beings who are attacked by it, also serves to multiply the maladies conjoined with it, exhibiting the severest complications and the most afflicting pictures; such, in my opinion, is the true and simple method of observing and analysing this affection of the hair, without there being any necessity for stopping, with M. de la Fontaine, at the fastidious divisions and subdivisions of species, into which every inventor has sought to introduce. I am rather better pleased with the sketch given by M. Alibert, of the external characters of plica, which he refers to three principal forms, and to their varieties; I qualify my opinion with respect to this writer, solely with a wish that he had excluded the denomination of species, the mathematical precision of forms which is not in nature, and the limitation of the varieties which may be innumerable, or at least furnish new forms to be described, such as the *round plica*, the *squamous plica*, &c.

The cause of plica is an important object, but the work in question glides over it very slightly, giving us only a few common places, without instructing us.

The author figures to himself a plicous matter hitherto unknown: taking his comparisons from the venereal, scrophulous, scorbutic and arthritic vices: he decides that the plica, by its proximate cause, is a "humour *sui generis*, viscous, acrid, and carried by the lymph into the hairs and nails, to be there deposited in a manner that may be regarded as critical." This *ex cathedra* language; this imperative tone, teaches me nothing, except that the motto of the coach is forgotten, the precept of Selle not followed, and the promises of M. de la Fontaine unperformed. We may also perceive that his reasons hesitate between doubt and affirmation. "It is not easy" he adds, "to determine what are the causes which produce this humour; for neither the air nor the water, nor even the food seems to contribute to it." What would the author bring us to, when, for these thirty years he has seen and attended thousands of patients, and yet leaves us so far behind with respect to the disease? This

This, in my opinion, is evidently the accidental impulse of a retrograde doctrine, adopted however by an excellent mind.

M. de la Fontaine ventures to say, "That we are equally liable to the plica whether we carefully comb our hair, or neglect it." This assertion is very vague, to say no more: it has never been hazarded by any observer. Admitting, however, the possibility of the fact, it is invariably true that combing the hair renders the affection much more rare, setting aside any other domestic causes that may provoke it. Can the author be ignorant that the state of abject servility inseparable from the lower classes in Poland, induces such a state of apathy, that the only combs they use are their nails, and these are only applied when the itching of vermin forces them to resort to scratching? Can he be ignorant that in the higher classes, among whom the plica is very rare, notwithstanding the singularity of some cases, cleanliness is most rigidly attended to.

On these points, I am in possession of authentic facts, because a physician, when he seeks to probe the source of the *trichoma* to the bottom, ought not to take advantage of any general charge of want of cleanliness, otherwise he is guilty of a voluntary neglect of the most simple appearances of the disease.

The article of the author on the cure of plica, subjoined to an appendix on the question of cutting off the hair, comprises 25 pages, of which I have already in another place given the following summary: "The plan of treatment refers to the specific virtues assigned to antimony, and gives conjectures as to the eruption, explosion, crisis, metastasis, and other phantoms of the same kind. In all this M. de la Fontaine conforms to the established ideas; in what he has written we perceive a very intelligent mind subjugated by the errors of others."

M. Franche, author of a large work on medical police, and of the excellent *Epitome de Morbis*, resided at Posen two or three years before I did, and nearly for the same space of time. Many cases of plica occurred in his practice, on that occasion; he succeeded in eradicating the disease as often as it occurred, by administering the remedies indicated by the kind and degree of cachexia under which the patient laboured. This mode of practice so naturally resulting from rational medicine, obliterates the five-and-twenty pages of M. de la Fontaine, and every romance of the same description. The true system on the subject of plica, assigns its commencement to an extreme neglect of cleanliness, and to the filth which is consequently propagated to all around the same fire-side: the same system, proceeding afterwards from cause to effect, calls our attention to other circumstances prejudicial to health. These last, last, on the contrary, are regarded as the chief objects by the inhabitants and medical practitioners of Poland: they are referred to in an inverse ratio to an alleged specific virus, the *materia trichomatica* of the Germans. M. de la Fontaine, and several others before him, have made this the basis of an universal pathology. Here we have the unavoidable mistake, resulting from substituting effect for cause.

M. de la Fontaine thus commences his chapter on the method of cure: "Sometimes, as I have already mentioned, we see the plica developed without having been preceded by any disagreeable accident, separated from the head and replaced by new and sound hairs, without the patient having recourse to any remedy." What he had *already mentioned* is to be perused at p. 17. of his work, in the following words: "Frequently," (instead of *sometimes)* "the plica declares itself without being preceded by the slightest indisposition, (instead of *any disagreeable accident.)* This fact, although it does vary in the mode of expressing, it is sufficient to prove to the reader, that the majority of the Polish nation never have the plica; a great part of the minority in this calculation have this disease as an external affection only, and which may be avoided by attention to cleanliness; the most of these last individuals are in good health, both before and during the disease; it would be ridiculous to assign the smallest trichomatic vice to them. There remains, therefore, but a small number of miserable beings who may be considered as really diseased, and to whom we must apply either by supposition, or conditionally, the assertion of the author, namely, that "this affection is not always equally mild, and that most commonly it requires both internal and external applications." The reader is left to judge if the trichomatic vice is still necessary.

"The curative treatment ought to be appropriated at every stage of the disease, for it does not exhibit itself in the same manner at its origin, before its critical passage into the hair, as during and after this passage." A reader, even although not very nice, will ask what is the meaning of this origin and critical passage, of which he sees nothing; he would prefer the actual representation to the explanation of the thing. The study of the best ascertained virulent diseases, such as syphilis, small-pox, &c. furnishes visible proofs of their origin, of the developement of their symptoms, and of the crisis by which they are terminated. Why should there be in *plica* a progress altogether extraordinary and quite metaphysical? Where is the proof of a critical effect, when there is nothing but what is accidental or symptomatic, and when the most palpable observation, that of the hair which is clotted, clotted, is sufficient, since it falls off under the cognizance of our senses? M. de la Fontaine continues: "When the plica shews itself, we ought exclusively to apply ourselves to soften the viscous, gluey, acrid, and irritating matter, and to render it fit for passing into the hair." Here then we have the thickening of a viscous and gluey matter, which may mechanically retard the critical transition just spoken of; but the plica does not exist the less for this, and it must be paid attention to: "We obtain this object (that of softening the viscous matter) by the use of dissolving, diluting, saponaceous and emollient plants, such as burdock, chicory, bitter-sweet, sassafras, guaiacum, &c. Sometimes these methods are sufficient for determining the crisis; but in most cases they ought to be united with the following: extract of henbane, hemlock, flowers of sulphur, calomel, Plumer's alterative powder, golden sulphur of antimony recently prepared, Theden's antimonial tincture, and antimonial lozenges. Antimony acts against plica with almost as much energy as mercury does against the venereal disease. If it be summer time, the patients ought to be put on a diet consisting of the juice of vegetables mixed with broth or whey; or rather this last liquid ought to be prescribed instead of the decoction of the plants above mentioned." All this pharmaceutical regimen still admits of the plica being still seen in full vigour without any check; this is not a treatment of the disease; but let us follow the author.

"If the morbific principle, by the use of these different means, be not disposed to pass into the hair, as is the case with the viscous exudations from the head; he may have recourse to sudorifics, such as the spirits of mindererus, amber, volatile alkali, Dover's powders, and henbane mixed with camphor, &c. The formulæ given at the end of this work have almost always attained the objects I had in view." Here the same reader will not think himself obliged to connect with the lazy theory of the morbific principle disposed to pass into the hair, the symptomatic consequence of the sweats of the head which take place spontaneously from heat, vermin, the filth of the plica, and the action of the coverings used on the head: the following is the real state of the matter. In sound reason, the defective volatile preparations, and the formula enumerated by the author, have no more therapeutic relation with the phenomenon in question, than the double series of preliminary remedies above detailed, and among which it would not be difficult to point out several empirical absurdities.

What M. de la Fontaine says on the subject of his experiments ments made with lycopodium is not very conclusive; the employment of the substance formerly recommended against the *trichoma*, brings to our recollection those popular remedies accredited *post hoc* and *propter hoc*, in diseases subject to intervals of suspension, or of long intermission. The internal exhibition of the powder of lycopodium, its external application, and the employment of the plant in substance as a bath, and in fomentation, according as the disease has appeared to be interrupted, have led some persons astray; but the plica has not the less ceased to re-appear as usual, amid the causes which never fail to produce it.

We are apprized that we cannot have recourse to the different remedies hitherto detailed, except when there is no fever: this ought to put his readers on their guard. "*If* fever manifests itself, and it is violent, we must have recourse to antiphlogistics, and even venesection as a precaution. This however must be practised with circumspection. *If* obstructions are accumulated in the primæ viæ, we ought to administer digestives, cooling evacuants, and vomits, taking care however not to prescribe evacuants, except in urgent cases, and to be cautious in them all." The fever, or the various types of fever, are among the number of conjoint and remittent diseases which ought to be exhibited in the same sphere in which we see the *trichoma*. The reader will judge that the fever is without doubt an incident which throws discredit on the system of the author, and which a true physician would not pass over so slightly as with antiphlogistics, bleedings, digestions, vomits, and evacuants, if there is a complication of obstructions, &c. But let us follow the author through the rest of the chapter.

"As the small-pox cannot declare itself without fever; in the same manner without this symptom, the plica cannot throw itself into the hair. We ought, therefore, to place no obstacle in the way of this fever, which is so necessary on producing the crisis; we ought rather cautiously to avoid disturbing nature in her progress; we ought to increase the fever if it be too weak, and weaken it if it be too violent; in short, to keep it at a proper degree, in order that the crisis may take effect; at this stage, the greatest attention is requisite on the part of the physician."

The reader, astonished at the common places, so elegantly jumbled together in the above article, will no doubt say, that the comparison between the small-pox and the trichoma is not a very happy one; the fever, preparatory to the variolous eruption, is a constant phenomenon; it may be regarded as a necessary element of the exanthematous, fermentation; mentation; but the fever which M. de la Fontaine wishes to establish as a condition, without which the plica cannot be thrown into the hair, while this plica already exists, and is visible even in the eyes, is a mere chimera. Has he not told us immediately before, that *sometimes*, nay *frequently*, we see the trichoma formed without the smallest indisposition; but small-pox admits of no such exceptions. The parallel he wishes to draw, therefore, proves the absurdity of the observation, and there is neither occasion for a fever nor a crisis in plica. *Hypothesis mentita est sibi.*

If I should have incurred the reproaches of my readers for not having sufficiently detailed in this extract the trichomatic symptomatology of M. de la Fontaine, and for having called the whole of it in question, they will now see that I was well founded in my scepticism: for if this pretended fever was a capital symptom, and consequently to be described before describing the method of cure, it would be as essential to the invasion of the plica as the usual fever is in small-pox. Now the author has not said a word on this subject; and I have made a most rigid scrutiny to discover if he has. It gives me pain to be compelled to notice the numerous assertions hazarded at pleasure, and soon invalidated or contradicted in subsequent parts of the same book. The reader will be enabled to judge of the truth of what I say from a perusal of the present analysis.

Notwithstanding the length to which I have already brought my observations, I must still claim indulgence for a few more quotations: "The crisis, as I have already said, is frequently decided suddenly; but frequently also it does not appear until some days, weeks, or even months have elapsed." Here we have the word *frequently* twice in conflict for two different events, one of which seems to be of more rare occurrence than the other. If we turn to what he has already said, we find these words: "The plica sometimes is not manifested until after several weeks, several months, *or even several years*." This version affords us a little more latitude than the foregoing. "All this depends," the author adds, "on the disposition of the humours, and on the intenseness of the fever."

Nothing certainly can be more luminous, or more conclusive than this solution—"If the patient be already weakened by anterior accidents or by age" (he might have said, also, by the abuse of remedies, or by imaginary modes of treatment) the fever is commonly insufficient to produce the crisis; we ought, therefore, to increase the strength of the patients patients by generous food, such as rich soups, chocolate, wine, &c."

What are we to make of this galimaufry, so aptly called by Horace, the *voces inopes rerum?* What becomes now of the comparison of plica with small-pox, so far as the *sine qua non* condition of fever is concerned. Let us take the most reasonable supposition, namely, that the patient being so weakened, that he can support nothing. The crisis, the fever, which ought to come on, do not make their appearance; this does not please M. de la Fontaine, however, and yet he seems disposed to renounce his theory, when he endeavours to strengthen his patient by good analeptics. The expedient is sage, and would no doubt be agreeable to all plicous patients; but unfortunately, it is likely to remain on paper only, as the disease is confined to the poor, who cannot command the objects of luxury thus liberally prescribed.

Throughout the remainder of the description of the method of cure, there are perpetual considerations, in consequence of the chimerical phenomena adopted by the author: all kinds of symptoms, accidents, and anomalies of every possible disease, are referred to the phamtom of a trichomatic habit. If it be a merit to accumulate more sophisms than any other writer on the trichoma, M. de la Fontaine has a claim to great honours; he affirms the pro and the contra indiscriminately; he promises success on all cases, and yet gives us proves of it in none. The grand consequence of his ideal doctrine is to refer every thing to plica, and to its filthy concomitants; by his perpetual recurrence to the latter, he exhibits the true origin of this dreadful disease; an origin which he refuses to assign to it; in his impatience to obtain the crisis of this nasty disease, he does not scruple, to the disgrace of science, to provoke it by artificial means, namely, by inoculation. This is the very crisis of insanity!

A long article by M. de Lafontaine, on cutting off the hair in plica, is written in the same spirit of hypothesis and error. If, according to him, "in an infinite number of plicas the hair has been cut off without the smallest disease resulting," why is this salutary measure not made the basis of a most simple and appropriate treatment? Professor Franche, Dr. Hennich, and I have repeatedly adopted this practice with uniform success.

I shall now conclude with briefly alluding to the twenty cases of plica with which the work terminates, and present my readers with a few cursory reflections suggested by their perusal.

With respect to his first case, I can never admit of peripneumonia being attended with plica as a matter of course. The subject of the second case is a female, who was so deluged with medicines for rheumatism that she fell into severe convulsions. The disposition to plica here was extremely hypothetical: the irritation was provoked at the roots of the hair by profuse sweats, and by the hair being clotted. There is nothing in this case to prove that rheumatism and plica are in any way connected. The third case is in my opinion insignificant; the trichoma is no preservative against the effects of drunkenness. The fourth case refers to a paralytic patient who recovers the use of his limbs after a copious sweat: was it indispensible that he should also have the plica at the same time in the hairy part of his body? Strengthening medicines completed the cure, as the author informs us. I have deduced from the fifth case, some consequences respecting the deformity in the nails of the toes; and also observed in gouty persons, without being ascribed to a plica polonica. I have particularly remarked in the sixth case, and in the observation subjoined to it, the abuse of the *post hoc*, propter hac, and the aptitude to deduce general laws from particular appearances. The following is a palpable truth of all the accidents which occasion trichoma; one of the most frequent is that of acute, eruptive, and other diseases, in which the patients are confined in infected beds drowned in those copious sweats; the dirt and danger of which is despised by the Polish physician. The seventh case, of chronic ophthalmia, in which the plica was created in the toes, is one of the most marvellous: any where but in Poland, the practitioner would have resorted to a more sagacious treatment. The same observation applies to the eighth case, of a similar kind of disease; and the author concludes his remarks by an avowal equally ingenuous and expressive: —"I have sometimes seen myself," he says, "obliged to lavish my care during a whole year without attaining my object. I have even seen cases, in which all that I did was fruitless; the disease remaining constantly obstinate." The hypopion related in the ninth case, seems to do particular credit to the excellent surgical treatment of M. De Lafontaine; every thing is due to art, but nothing to the trichoma, or to the pretended specifics. We see evidently the good effects of a blister on the head, and not the critical virtue of the *trichoma* in the treatment of the four cases of nyctalopia, in number ten. The same took place in the eleventh case. I have nothing to say of the twelfth case, in which is described an unsuccessful operation, or a cataract produced by an accident quite distinct from plica. The two following cases (13 and and 14) describe more fortunate extractions of cataracts, under circumstances which do credit to the skill and dexterity of the oculist, but all this has nothing to do with the innate trichomatic virus, the laws of which have been so dogmatically laid down by the author. The 15th case also concerns the oculist only. It is not difficult to trace in the four succeeding cases a principle of cachexy, particularly of the evil to which the plica is merely accessary.

The twentieth and last case relative to a plica of the sexual organs in a woman in child-bed, affords me an opportunity of discussing a fact which M. de la Fontaine has several times touched on, namely, the case in which the detached trichoma hangs from the sound and fresh growing hair. It is then that he consents to extirpate it; and I fully agree with him, with this addition, that the case is much more general than he seems to insinuate; for every plica is composed alternately of hairs, which clot, and are broken or fall off, while new hairs sprout up irregularly from the same surfaces, and these last finish by becoming bald in whole or in part. The effects of the trichoma from that moment adhere only to a very few roots in comparison wi h the size of the surface of the body from which they issue, and which sometimes excite an acute pain on the slightest touch. Let it be remarked, however, notwithstanding all that has been said to the contrary, that the skin is never in the slightest degree diseased: I have always found it in the natural state.

Before giving the formula, to which I have nothing to say, and previous to the description of the plates, in which the artist's pencil has but ill represented the irregularities of nature in this disease, the author occupies a few pages with an account, communicated to him, of a wolf-dog which had been attacked with trichoma; the details appear to me to be extremely marvellous, I leave them to the reflections of the reader; he may compare this case with what I have already said respecting the dog at Posen, and the Lion at Hesse Cassel* in which there was no prodigy. Such is the singularity, the affectation, and the doubtful impression of most dogmatical cases, in which the mind of the narrator delights in equivocal abstractions, that we are continually inclined to ask, *if the fact be true?* This is not the case with the Hippocratic method, in which reason is regulated by the faithful report of the senses. It is thus that Morton persuades his readers;—Riviere also is admirable for his details of cures; he describes

* See No. 130 of this Journal.

 and and relates more than he reasons. This is the true touchstone of medical observation and the genius of Barthez has dictated its last precepts.

Whatever may be my opinion of the present work of M. de la Fontaine, I cannot refrain from paying homage to those talents which have so long and so deservedly placed him in the highest ranks of the profession.

---

*A Conspectus of the London, Edinburgh, and Dublin Pharmacopœias, &c. &c. &c.* By Robert Graves, M. D. F. L. S. 4th Edit. London, 12mo. 1810. pp. 135.

In the earliest period of the healing art, the instruments of medicine were few in number, their powers were ill understood, and their application in the cure of disease absurd and inefficacious. Another period saw the Materia Medica loaded with a confused abundance of articles, the employment of which was guided by theories that had little regard to the laws of nature, and were often in direct contradiction to plain matter of fact. In a third period, the Physician returning to the simplicity of truth, selected, arranged; and pruned, with a bold hand, inert redundancies.

The latest effort to improve the condition of this essential branch of the medical art, has been the Pharmacopœia of the London College of 1809. The Pharmacopœias of Edinburgh and Dublin had previously been revived. Some disagreement still, however, exists in the nomenclature of these authorized collections. A few years since, this little volume undertook to bring into a perspicuous form, and in a size adapted to the pocket, the sometimes discordant materials of the preceding works. It had then our fresh approbation; and many opportunities have confirmed its practical utility. This new edition, which the alterations made in the London and Edinburgh Pharmacopœias have called for, we cannot doubt will prove equally useful.

As a specimen of the execution of this convenient volume, we insert the following new articles.

Arsenici Oxydum (i. n.) Præparatum. Prepared white arsenic; tonic; in intermittents, periodic headachs. leporous affections, &c. (See Lig.) in cases of deleterious or poisonous effects from it, recourse should be had to a free use of mucilages and milk, particularly the former, to sheath the stomach, and to a solution of sulphurat of potash as a corrective. Externally escharotic; against cancerous sores, arsenic gr. iv. water ℔ij, or arsenic ʒss—℈ij, cerate of sperma ceti and hogs lard, each ℥ss.

Liquor Arsenicalis. L. Arsenici oxidi præp. in pulverem subtiliss. triti, potassæ subcarbonatis extartaro, sing. gr. lxiv. Aquæ dist. ℔j. Boil them together in a glass vessel, until the arsenic is dissolved. When the solution is cold, add Spir. Lavend. comp. ℥ss, and as much distilled water as will make the whole equal to a pint. Tonic, M v ad M x bis terve die, diluted in thick gruel.

Belladonnæ Folia. Deadly nightshade leaves. Narcotic, diaphoretic, diuretic, resolvent; in cancers, schirrhus, mania, epilepsy, &c.

&c. in infusion gr. ss. ad gr. v, or more, bis die. See *Extract.* From the uncertain operation of this medicine, it is prudent to begin with small doses, and to increase them gradually, according to their effects. Also externally against cancer, schirrhus, &c.

EXTRACTUM BELLADONNÆ. L. E. gr. ½ ad gr. iij, or more, bis terve die.

HUMULI STROBILI, (orum, m.) hops. L. Tonic, sedative, aromatic; in atonic gout, &c. See *Extr. & Tinc.*

EXTRACTUM HUMULI. L. Tonic, sedative, gr. v ad ℈j, bis, ter, quaterve die.

TINCTURA HUMULI. L. (Humuli strobili ℥v. Spir. ten. ℔ij. Digest for fourteen days and strain.) Tonic, aromatic, ʒj ad ʒij.

While many a splendid volume moulders in dusty obscurity on the bookseller's shelf, this modest and unpretending compilation will find its way into the pocket of every practitioner.

——————To know
That which before us lies in daily life
Is the prime wisdom.

---

*An account of Spina Bifida, with remarks on a method of treatment proposed by Mr. Abernethy. By* THOMAS VERNEY OKES. *Cambridge printed; 8vo.* 1810. *pp.* 39.

IT is the fate of all human invention to have in it the alloy of imperfection. The practice of medicine, and even the art of surgery, which is vaunted to have more of precision and certainty than its congenors, partake ol this penalty. The quaint appellative, *noli me tangere*, given to a species of harpers, has been too restricted. The course of actual practice has not unfrequently presented original conformation, and sometimes morbid alteration, which should have affixed to them *in terrorem, noli me tangere*; of this class is *Spina Bifida.* A long acquaintance with the steady judgment and practical skill of Mr. Okes, made us take up his pamphlet with a predilection in its favour. For it is to such men, though, perhaps, unskilled in the art of bookmaking, we are to look for the lessening of human misery, by the improvement of medical science. If in the present instance the experience of this gentleman has enabled him only to give negative instruction, we hope to see him unlock the stores of practical knowledge which many years of application must have accumulated.

The just reward of diligence and eminent abilities, is found in that currency of opinions which arises out of reputation. But sometimes has this been injurious, by stamping a value on erroneous doctrines. The deservedly high character of Abernethy, induced Mr. Okes to investigate the nature of *Spina Bifida*, a disease of which Mr. Abernethy had published some cases, and advised a method of treatment from analogy, extremely hazardous, if not always fatal. "From the success with which the evacuation of the matter in lumbar abscess, by means of the trocar, has been attended, Mr. A—— was induced to recommend a similar process in *Hydrorhachitis*:" and also to advise pressure upon the punctured saculus to promote absorption. A number of cases are cited and arguments employed by Mr. Okes to shew the dangerous dangerous tendency of this practice ; and from them are deduced the subsequent corollaries.

1. If an hydrorhactic tumour should ulcerate or slough, so that an opening be made, the patient will inevitably die.

2. If the contents be evacuated by trocar or lancet, and the opening be left unclosed, the patient will die.

3. If a ligature be applied round the base of the tumour, the patient will die.

4. If the contents be evacuated by trocar, and the opening be closed and healed by the first intention, the sac will not contract so as to prevent a fresh accumulation of lymph, but it will be speedily and repeatedly reproduced, and no advantage will be gained by the operation.

The description of *spina bifida,* with which the pamphlet commences, we insert with the view of disseminating practical knowledge.

"The disease which is usually called spina bifida, sometimes divided spine, spinola, and more properly hydrorhachitis, proceeds from a mal-conformation of the spine, and originates with the fœtus in utero. The spinal processes, and sometimes the lateral processes of some of the vertebræ are wanting, by which means there is a longitudinal opening of the bony cylindrical channel which contains the medulla spinalis. This deficiency sometimes takes place in one part of the spine, and sometimes in another ; but I believe more commonly in the vertebræ of the loins, or in the os sacrum, than in any other part. It is remarkable that where there is this defect in the formation of the bones, there is generally a corresponding defect of the integuments over that part of the vertebræ ; for the cutis and membranæ a diposa are wanting for a considerable space, and the opening will be found covered only by a very thin, teuder skin, and so transparent that the contents may be seen through it ; and their covering is doubtless a production of the duramatral coat of the medulla spinalis. Sometimes, however, the common integuments are perfect, and of their natural thickness and opacity. From the differenee in the coverings of the tumour it may be distinguished into two sorts, the transparent and opake. The tumour which is formed contains a clear fluid, like the lymph found in the lateral ventricles of the brain, and hence the disease has required the name of hydrorhachitis. There is another shape under which this disease appears, that is when there is no deficiency in the formation of the vertebræ themselves, but where the fluid and the duramatral coat are forced through a small separation of the spinous processes, producing a tumour of the back with a small base. This has given rise to a proposal for what I consider a dangerous mode of practice ; for from the smallness of the base of the tumour practitioners have been induced to suppose that it is easily removeable : experience, however, has proved that this cannot be safely attempted."

Cases from Sulpius, Ruysch, and Morgagni, with plates ; and observations on the analogy which *spina bifida* has with diseases intimately connected with the coverings of the brain, illustrate and enforce the author's objection to Mr. Abernethy's proposed practice. For several interesting histories of this disease we refer to the preceding volumes of the Medical and Physical Journal.

Though

Though we have no extravagant liking for the dainty phrases, piquant remarks, and holiday terms of modern authorship, we would be well pleased to see a judicious practitioner sufficiently master of composition, to place his observation in the best light; and we recommend to Mr. Okes, with a sincere regard for his useful talents, that attention to style and arrangement proper to give force and character to his future productions.

---

*Letters on Professional Character and Manners: on the Education of a Surgeon and the Duties and Qualifications of a Physician: addressed to James Gregory, M. D. Professor of the Practice of Medicine in the University of Edinburgh.* By JOHN BELL, Surgeon. Edin. 1810. pp. 636.

IT must give pain to every true lover of the medical profession to observe the violent disputes which have for so long a period subsisted and disgraced the first school in Europe. These disputes do not arise from the discussion of any scientific question, nor tend to the perfection or amelioration of the healing art, but are mere personal contentions for posts and places, for employment and emoluments. It is well known to our readers that the waters of strife were first let out a few years ago, on the new system of electing surgeons to the Royal Infirmary in that city being first adopted, and much rancorous altercation then took place, in which the celebrated Professor to whom these letters are addressed, took no inconsiderable share. Whatever might have been thought at the time, of the motives which induced him thus keenly to espouse one particular side of the question, the purity of them must now be much suspected, if Mr. Bell has in the present publication detailed the circumstances fairly and candidly. The calm which succeeded this storm was of short duration, and but portended a renewal of the tempest with increased violence. An occasion soon arose, a proposition submitted to the college of Physicians by the junior members of it, for allowing them to engage to a limited degree in the practice of Pharmacy, called forth a bitter opposition to it from those who foresaw and dreaded the deep disgrace in which the whole college would be involved; among these our Professor maintained a conspicuous station, and with his accustomed impetuosity thundered out his denunciations, and poured forth a torrent of abuse which reached every individual opposed to him. Into the merits of the question we do not propose to enter, but to leave the consideration of it to those who are more immediately concerned in its determination; it is vain, however, to look for any cool and temperate discussion of it, in the present inflamed and irritable state of the parties, and we fear that every future step taken in the business will only be the means of adding fuel to the flame.

That the conduct of the Professor in this affair has not been free from blame, we cannot but believe, and Mr. Bell has, with no little asperity and apparently with some reason, undertaken to reprehend him.

"That Dr. James Gregory has traduced my professional and moral reputation, is most true: it is the most trivial perhaps of his offence, that which the public will most easily pardon, and which I was inclined, from pride pride and conscious rectitude, to pass in silence. Seven years have elapsed, and more, since he sought to ruin my good name, and concluded a defamatory quarto of five hundred and thirteen pages with this memorable warning to the inhabitants of this my native city. 'Any man, if himself and his family were sick, should as soon think of calling in a mad dog, as Mr. John Bell, or any who held the principles he professes.' My principles, my feelings, my professional talents, and my integrity, may easily find more partial judges than Dr. Gregory. If I have made any uncharitable conclusion concerning his, it is one which conveys a compliment to his prudence. He wrote, published, distributed, this memorable and calumnious essay on my poor talents, not merely for the gratification of his own malicious propensities, but for behoof of those who could reward them. By the patience with which I endured calumnies, which I never wanted spirit or talent to repress, I showed how willing I was that contention should cease, and the profession in this city be restored to a share of public esteem. But this foul attempt against individual reputation, was only the prelude to universal aggression, and my silence an immunity for past, and an invitation to greater offences. Volume after volume has issued from the press, fraught with defamation, ribaldry, and obscenity; and tales and jests, portentous to our much honoured profession and to the fame of the medical school."

These letters are written with no inconsiderable humour, but they are extended to too great a length; and while reading them we cannot escape the painful recollection of the disgrace which attaches to the profession from the circumstances they disclose. On the relation of an operation performed some time since in the Infirmary, we forbear to make any observation whatever.

