# Errata

**Published:** 1810-11

**Authors:** 


### ERRATA.

Page 340, line 23, *for* revived, *read* revised.

line 27, *for* fresh, *read* full.

line 35, *for* lig. *read* liq.

341, line 26, *for* harpers, *read* herpes.

342, line 3, *for* hydrorhactic, *read* hydrorhachitis.

line 45, *for* Sulpius *read* Tulpius.

